# Human Single-Chain Antibodies That Neutralize Elastolytic Activity of *Pseudomonas aeruginosa* LasB

**DOI:** 10.3390/pathogens10060765

**Published:** 2021-06-17

**Authors:** Sirijan Santajit, Thida Kong-ngoen, Manas Chongsa-Nguan, Usa Boonyuen, Pornpan Pumirat, Nitat Sookrung, Wanpen Chaicumpa, Nitaya Indrawattana

**Affiliations:** 1 Department of Microbiology and Immunology, Faculty of Tropical Medicine, Mahidol University, Bangkok 10400, Thailand; sirijan.sa@wu.ac.th (S.S.); thida.kon@mahidol.ac.th (T.K.-n.); pornpan.pum@mahidol.ac.th (P.P.); 2 Department of Medical Technology, School of Allied Health Sciences, Walailak University, Nakhon Si Thammarat 80160, Thailand; 3 Faculty of Public Health and Environment, Pathumthani University, Pathum Thani 12000, Thailand; manas.cho@mahidol.ac.th; 4 Department of Molecular Tropical Medicine and Genetics, Faculty of Tropical Medicine, Mahidol University, Bangkok 10400, Thailand; usa.boo@mahidol.ac.th; 5 Center of Research Excellence on Therapeutic Proteins and Antibody Engineering, Department of Parasitology, Faculty of Medicine Siriraj Hospital, Mahidol University, Bangkok 10700, Thailand; nitat.soo@mahidol.ac.th (N.S.); wanpen.cha@mahidol.ac.th (W.C.); 6 Biomedical Research Incubator Unit, Department of Research, Faculty of Medicine Siriraj Hospital, Mahidol University, Bangkok 10700, Thailand

**Keywords:** elastase, elastolytic activity, human single-chain antibody, HuscFv, *Pseudomonas aeruginosa*, LasB, phage display technology

## Abstract

LasB (elastase/pseudolysin) is an injurious zinc-metalloprotease secreted by the infecting *Pseudomonas aeruginosa*. LasB is recognized as the bacterial key virulence factor for establishment of successful infection, acquisition of nutrients, dissemination, tissue invasion, and immune modulation and evasion. LasB digests a variety of the host tissue proteins, extracellular matrices, as well as components of both innate and adaptive immune systems, including immunoglobulins, complement proteins, and cytokines. Thus, this enzyme is an attractive target for disarming the *P. aeruginosa.* This study generated human single-chain antibodies (HuscFvs) that can neutralize the elastolytic activity of native LasB by using phage display technology. Gene sequences coding HuscFvs (*huscfvs*) isolated from HuscFv-displaying phage clones that bound to enzymatically active LasB were sub-cloned to expression plasmids for large scale production of the recombinant HuscFvs by the *huscfv*-plasmid transformed *Escherichia coli*. HuscFvs of two transformed *E. coli* clones, i.e., HuscFv-N42 and HuscFv-N45, neutralized the LasB elastolytic activities *in vitro*. Computer simulation by homology modeling and molecular docking demonstrated that antibodies presumptively formed contact interfaces with the LasB residues critical for the catalytic activity. Although the LasB neutralizing mechanisms await elucidation by laboratory experiments, the HuscFvs should be tested further towards the clinical application as a novel adjunctive therapeutics to mitigate severity of the diseases caused by *P. aeruginosa*.

## 1. Introduction

LasB protease, known also as pseudolysin or elastase, is one of the toxic extracellular enzymes secreted by the infecting *Pseudomonas aeruginosa via* the bacterial type II secretion system [1,2]. This protease belongs to the M4 thermolysin family of zinc-dependent neutral metalloendopeptidases [3,4,5,6,7,8]. LasB causes destruction of the tissues and degradation of a variety of proteins of the infected mammalian hosts including elastin (elastolytic activity), casein, types III and IV collagens, fibronectin, etc.; all for establishment of successful bacterial infection, further invasion and dissemination, and nutrient acquisition [9,10,11,12]. Besides, the LasB promotes the *P. aeruginosa* infection by modulation and regulation of the host innate and adaptive immune responses. For example, LasB lyses fibronectin to expose the host receptors for the bacterial attachment; digests serum-α1-proteinase inhibitor, surfactant proteins A and D, and bronchial mucosal proteinase inhibitors to disrupt the respiratory epithelium and destroys the ciliary function; digests human immunoglobulins (IgG and IgA) and complement proteins; represses gamma interferon and tumor necrosis factor; disrupts alveolar macrophage activity by downregulation of reactive oxygen species generation to interrupt the bacterial killing [13,14,15,16,17]. The enzyme has also strong hemorrhagic activity and muscle destructive effects [18]. It involves in pathology of a variety of diseases caused by *P. aeruginosa*, including lung infections by increasing lung permeability, impairment of apoptotic cell clearance in cystic fibrosis and bronchiectasis, and cleavage of surfactant proteins [17,19,20,21]; chronic ulcer by degrading human skin proteins and wound fluids [22]; and corneal infection by causing corneal liquefaction, which can damage the vision functions [23].

Because the LasB protease plays key pathogenic roles during *P. aeruginosa* infection, the enzyme is one of the potential therapeutic targets for mitigation of the pseudomonal disease severity [24,25,26]. Previous data have shown that rabbits infected with a Δ*las*B mutant strain displayed decreased severity of *P. aeruginosa*-mediated corneal ulceration [27]. Deletion of the *las*B gene resulted in less invasive *P. aeruginosa* infection in both mouse and *Caenorhabditis elegans* models, when compared to the infection caused by the wild-type strain [28,29]. Several antimicrobials and inhibitors have been used to block the synthesis of *P. aeruginosa* proteins (including LasB) that are crucial for bacterial survival and pathogenicity, i.e., kirromycin, pulvomycin, macrolides, clindamycin, chloramphenicol, aminoglycosides, tetracyclines, and synthetic oxazolidinone such as linezolid indole dipeptides, benzimidazole amidines, 2-arylbenzimidazoles, *N*-substituted imidazoles, guanidines, and fusidic acid [30,31,32]. Moreover, several inhibitors have been developed as the LasB inhibitors including 10-phenanthroline-5,6-dione (phendione) and its derivatives, hydroxamate-based MMP inhibitors, and *N*-mercaptoacetyl-Phe-Tyr-amide [24,33,34]. Nevertheless, *P. aeruginosa* has multiple strategies to resist the antimicrobial drugs and becomes a member of “ESKAPE” (an acronym for the group of six highly virulent and antibiotic resistant Gram-positive and Gram-negative bacteria that include *Enterococcus faecium*, *Staphylococcus aureus*, *Klebsiella pneumoniae*, *Acinetobacter baumannii*, *Pseudomonas aeruginosa*, and *Enterobacter* species). The LasB chemical inhibitors tend to be toxic to mammalian cells, which limits their therapeutic usage. In this study, engineered human monoclonal single-chain antibodies [HuscFvs], which are small molecules [consist of only variable heavy chain domain (VH) and variable light chain domain (VL) linked together via a (Gly_4_Ser)_3_ peptide; VH-linker-VL] that bind to and neutralize elastolytic activity of the *P. aruginosa* LasB were generated using phage display technology and a human single-chain antibody (HuscFv) phage display library as an *in vitro* biological tool [35,36,37]. The LasB-neutralizing HuscFvs should be tested further toward clinical application as an adjunctive therapeutics for *P.* *aeruginosa* infection.

## 2. Materials and Methods

### 2.1. Preparation of Native LasB 

*Pseudomonas aeruginosa* strain PAO1 was cultured in 250 mL of Luria-Bertani (LB) broth at 37 °C with shaking aeration for 18 h. The cell-free culture supernatant was collected after centrifugation at 15,000× *g*, 4 °C, 30 min. Purification of the native LasB (nLasB) from the culture supernatant was carried out using ammonium sulfate precipitation and DEAE-Sepharose column chromatography. In brief, the proteins in the culture supernatant were subjected to gradient ammonium sulfate precipitation. Firstly, 250 mL of the culture supernatant was added with 75 g of ammonium sulfate powder to yield 30% saturation. After the precipitate was removed by centrifugation at 15,000× *g*, 4 °C, 30 min, 200 g of ammonium sulfate powder was added to the supernatant to 80% saturation. The precipitate was collected by centrifugation as above, resuspended in 10 mL of 20 mM Tris-HCl buffer, pH 8.5, and filtered through a sterile 0.45 µm syringe filter. The preparation was dialyzed against the same buffer using Amicon^®^ Ultra 4 mL 3K centrifugal filter devices (Merck Millipore, Darmstadt, Germany) and further purified by using DEAE-Sepharose Fast Flow resin (GE Healthcare Life Sciences, Sweden). The protein solution was loaded onto the DEAE column pre-equilibrated with 20 mM Tris-HCl, pH 8.5. After allowing the proteins to bind to the resin for 1 h, the column was washed with the same buffer to remove the unbound proteins; then, the target enzyme was eluted by applying a linear gradient of NaCl (100−600 mM). Each collected fraction was analyzed by sodium dodecyl sulfate-polyacrylamide gel electrophoresis (SDS-PAGE) and Coomassie Brilliant Blue G-250 (CBB) staining. The fractions containing the nLasB were pooled and dialyzed against the same buffer. The protein quantity was determined by Bicinchoninic Acid Assay (BCA) using Pierce™ BCA Protein Assay Kit (TermoFisher Scientific, Waltham, MA, USA) following the manufacturer’s instruction. Different bovine serum albumin (BSA) concentrations were used for constructing the protein standard curve. Briefly, 25 µL of each BSA standard solution (20–2000 µg/mL) and protein sample were prepared in microplate wells (triplicate). Then, 200 µL of the working reagent (mixture of solutions A and B provided with the kit) were added to each well and mixed on a plate shaker. The plate was covered and incubated at 37 °C for 30 min. Absorbance at 595 nm of the content in each well was determined spectrophotometrically. The optical densities (OD) 595 nm of the standard protein dilutions were plotted against the known protein concentrations to construct the standard curve. Protein concentration of the unknown sample was extrapolated from the standard curve. The preparation was verified by LC/MS-MS and stored at −20 °C in small portions until use.

### 2.2. Determination of Enzymatic Activity of nLasB

The elastase activity of the purified nLasB was determined by the fluorogenic substrate assay using EnzChek Elastase Assay Kit (Invitrogen, Carlsbad, CA, USA), following the manufacturer’s instruction. The EnzChek Elastase Assay Kit provides a sensitive, convenient, and fast fluorometric method for measuring elastase (or other protease) activity or for screening the enzyme inhibitors in a high-throughput format. The substrate in this EnzChek kit is the highly fluorescein-labeled (BODIPY-FL-labeled) DQ-elastin conjugate where the fluorescence signal is quenched until enzymatic digestion to yield highly fluorescent fragments. The intensity of the emitted fluorescence signals correlates with the enzymatic activity. In the presence of the enzyme inhibitor, the fluorescence emission is reduced. For determining enzymatic activity of the nLasB, 25 μg/mL of the BODIPY FL-DQ conjugated-elastin substrate solution was added into individual wells of a microplate containing reaction buffer (provided with the kit). Different concentrations (25, 50, 75, and 100 nM) of nLasB were added appropriately into the wells, and fluorescence intensity of the content in each well was monitored using a fluorometer (Ex/Em = 485 ± 10 nm/530 ± 15 nm) at 37 °C for 2 h. Porcine pancreatic elastase, provided with the assay kit, was used as a positive control. 

The elastolytic activity of the nLasB was also determined by elastin Congo Red assay [13]. The basis of this assay is that digestion of elastin substrate (Congo Red-elastin complex) by LasB/elastase allows the Congo Red dye to be hydrolyzed and released from the dye-elastin complex, where the amount of the released dye directly correlates with the enzyme activity. In the presence of the enzyme inhibitor, the amount of the released dye is reduced. Various concentrations (12.5, 25, 50, 100, and 200 nM) of purified nLasB were added into wells of the reaction buffer (50 mM Tris-HCl, pH 7.5, and 0.5 mM CaCl_2_), containing 10 mg of Congo Red-elastin substrate (Sigma, St. Louis, MO, USA). The reaction mixtures were incubated at 37 °C for 2 h and terminated by adding EDTA. The clear supernatant after pelleting the undigested substrate by centrifugation was transferred into a new tube and optical density (OD) at 495 nm was measured. 

### 2.3. Phage Bio-Panning and Characterization of nLasB-Bound HuscFvs

The human scFv (HuscFv) phage display library used in this study was constructed previously [37]. Total RNA were extracted from lymphocytes of 60 healthy young adult volunteers and reverse transcribed to cDNAs. The cDNAs were used as templates for PCR amplification of immunoglobulin genes coding for variable heavy chain (VH) domains (*vh*) and variable kappa light chain (VL) domains (*vl*) by error prone PCR using human degenerate primers designed from all families/subfamilies of the human immunoglobulin genes. After amplification, the *vh* and *vl* sequences were linked randomly [via a nucleotide linker coding for a peptide (Gly_4_Ser)_3_] into *vh-linker-vl* (*huscfvs*) sequences before ligating to pCANTAB 5E phagemid vector downstream of the gene coding for a phage coat protein, P3. The recombinant *huscfvs*-phagemids were used to infect competent TG1 *E. coli* bacteria. The *huscfv-*phagemid-transfected-*E. coli* were grown and co-infected with a helper phage (M13KO7). The complete phage particles displaying HuscFvs of variable specificities as fusion proteins with the phage P3 on their surface and carrying integrated *huscfvs* in their genomes, were obtained from the bacterial culture supernatant. The HuscFv diversity (different epitope/antigen specificity) of this library was approximately 2.6 × 10^8^ [37,38]. 

Phage clones displaying nLasB-bound HuscFvs were selected from the HuscFv phage display library by phage bio-panning process [37]. Briefly, 0.5 µg of purified nLasB in 100 µL of 0.2 M sodium carbonate-bicarbonate buffer, pH 9.4, was added into a well of an EIA/RIA strip (Corning, NY, USA). After blocking the free spaces of the nLasB-coated well with Pierce^TM^ Protein-Free Blocking Buffer (ThermoFisher Scientific, Waltham, MA, USA), the HuscFv phage display library (50 µL) was added into the well and incubated. The unbound phages were washed away; the nLasB-bound phages were used to infect HB2151 *E. coli*. Then, the infected bacteria were spread onto selective 2× YT agar plates supplemented with 100 µg/mL ampicillin and 2%(w/v) glucose (2x YT-AG), and incubated at 37 °C overnight. The phagemid-transformed HB2151 *E. coli* colonies grown on the agar were screened for HuscFv genes (*huscfvs*) by direct colony PCR using pCANTAB-5E phagemid-specific primers [38]. The *huscfv*-positive *E. coli* clones were grown in 2× YT-AG broth at 37 °C until the OD 600 nm reached ~0.6 (about 3 h). The culture was added with 1 mM isopropyl-β-D-1-thiogalactopyranoside (IPTG) (Thermo Fisher Scientific) to induce the recombinant HuscFv expression and the preparation was incubated further at 30 °C for 5–6 h. Bacterial cells were harvested by centrifugation, resuspended in PBS, sonicated and centrifuged to remove the cell debris. The expressed soluble HuscFvs in the lysates of the phage-transformed HB2151 *E. coli* were tested for their binding to nLasB by indirect ELISA [38]. The diversity of the nucleotide sequences of the *huscfvs* was determined by Sanger sequencing. The *huscfv* sequences coding for soluble nLasB-bound HuscFvs of individual HB2151 *E. coli* clones were deduced, and their canonical complementarity-determining regions (CDRs) and immunoglobulin framework regions (FRs) of the VH and VL domains as well as the inter-domain linker peptides were worked out, based on the numbering scheme of Chotia and Kobat [39].

### 2.4. Large Scale Production of Recombinant LasB-Bound HuscFvs

For large scale production of the nLasB-bound HuscFvs, the pCANTAB-5E phagemids harboring *huscfvs* were sub-cloned individually into the pLATE52 expression vector (Thermo Fisher Scientific) using the ligation-independent cloning (LIC) protocol (aLICator LIC Cloning and Expression Kit 4; Thermo Fisher Scientific). The recombinant pLATE52-*huscfv* plasmids were transformed into JM109 *E. coli*. After colony PCR screening for the presence of *huscfvs* and DNA sequencing (Sanger) for the gene verification, the verified recombinant plasmids were introduced into NiCo21(DE3) *E. coli* (New England Biolabs, St. Albans, UK). A single colony of each transformed NiCo21(DE3) *E. coli* clone was cultured in 1 mM IPTG-conditioned broth to express recombinant HuscFvs. The recombinant HuscFvs were purified from the bacterial inclusion bodies (IBs) and refolded as previously described [40]. The concentrations of the refolded antibody preparations were determined using Pierce^®^ BCA Protein Assay Kit (Thermo Fisher Scientific). The quality and purity of the recombinant antibodies were analyzed by SDS-PAGE and CBB staining. The buffer of the refolded HuscFv preparations was changed to 20 mM Tris-HCl, pH 8.5, by dialysis; then, the preparations were concentrated using Amicon^®^ Ultra 4 mL 3K centrifugal filter devices (Merck Millipore), filtered through a 0.2 µm low-protein-binding Acrodisc^®^ syringe filter (Pall, Port Washington, NY, USA), and kept in 8% (*w*/*v*) glycerol at −80 °C for further use. 

The HuscFvs were subjected to circular dichroism (CD) analysis for determining their secondary structure and retested for nLasB binding by indirect ELISA to ensure their proper folding (retained specificity of the soluble HuscFv counterparts from the HB2151 *E. coli*).

For the CD analysis, the HuscFvs buffer was changed to 20 mM sodium phosphate buffer, pH 8.5, by dialysis and the protein concentration was adjusted to 0.1 mg/mL. The secondary structure of the refolded LasB-bound HuscFvs was determined using far-UV CD. The CD analysis was carried out on a JASCO J-815 spectropolarimeter equipped with a Peltier temperature controller system (Jasco, Tokyo, Japan). The CD spectra (190–260 nm) of refolded antibodies were recorded in a 1-mm path-length quartz cuvette at 25 °C and a scan rate of 50 nm/min. Three scans were averaged to generate the CD spectrum for each protein.

### 2.5. Fluorogenic Substrate Assay for Determining the LasB Neutralizing Activity of the HuscFvs 

The inhibitory effect of HuscFvs on the elastolytic (elastase) activity of the nLasB was assessed using fluorogenic substrate (EnzChek elastase assay kit; Invitrogen), following the manufacturer’s instruction. The elastase inhibition assay was performed by preparing the reaction mixtures (each mixture in triplicate): HuscFvs (2.5, 5, 7.5, and 10 μM) mixed with 25 nM nLasB (test mixtures), 25 nM nLasB mixed with 5 mM EDTA (positive inhibition control mixture), and 25 nM nLasB in buffer (negative inhibition control). All preparations were kept at room temperature away from the light for 15 min. Fifty microliters of 25 μg/mL the BODIPY-FL-labeled DQ-elastin substrate solution was added to each mixture and kept at room temperature in the dark for 30 min. The fluorescence emission was measured at 515 nm with excitation at 505 nm using a fluorescent microplate reader (Synergy H1 Hybrid Reader; BioTek, Winooski, VT, USA). The fluorescence signal increased after the substrate was digested by the active LasB (elastase). The signal is reduced in the presence of the enzyme inhibitor, compared to the reaction without the inhibitor. Three independent experiments were performed. 

### 2.6. Elastin Congo Red Assay for Determining the HuscFvs-Mediated Inhibition of the LasB Elastolytic Activity

HuscFvs-mediated neutralization of the nLasB elastolytic activity was evaluated also by the elastin Congo Red assay. The purified nLasB at 50 nM (100 µL) was mixed with various concentrations of HuscFvs (0.625, 1.25, 2.5, and 5 µM). Each mixture was incubated at 37 °C for 1 h. LasB alone served as negative neutralization control and 5 mM EDTA as positive neutralization control. Then the Congo Red-elastin substrate solution in reaction buffer was added and further incubated at 37 °C for 2 h with shaking at 250 rpm. The reaction was stopped by adding 100 µL of 0.12 M EDTA. The clear supernatant was collected by centrifugation at 15,000× *g* for 25 min and transferred to a new tube. Hydrolyzed Congo Red was determined by measuring the absorbance of the solution at 495 nm. Three independent experiments were performed. The net OD 495 nm of the each assay supernatant was obtained after subtracting with the background absorbance (OD 495 nm of the reaction mixture containing the indicated amount of HuscFv, substrate, and buffer without adding the nLasB).

### 2.7. Homology Modeling and Inter-Molecular Docking

The three-dimensional (3D) structure of wild-type *P. aeruginosa* elastase (PDB ID: 1EZM) was retrieved from the Protein Data Bank (RCSB PDB) [41]. The amino acid sequences of the HuscFvs were subjected to structural modeling using I-TASSER server service (http://zhanglab.ccmb.med.umic.edu/I-TASSER/; accessed on 12 September 2020) [42,43]. The qualities of the I-TASSER-predicted 3D structural models were subsequently refined in order to improve the local geometric and physical quality of the predicted 3D structure using ModRefiner high-resolution protein structure refinement (http://zhanglab.ccmb.med.umich.edu/ModRefiner/; accessed on 15 September 2020) [44], and fragment-guided molecular dynamics (FG-MD) simulation (http://zhanglab.ccmb.med.umich.edu/FG-MD/; accessed on 16 September 2020) [45]. The refined HuscFv 3D models and LasB 3D structure were subjected to intermolecular docking using the automated ClusPro 2.0 protein-antibody docking server [46,47,48]. All antigen-antibody complexed models were analyzed and visualized using Discovery Studio 3.5 and PyMol software (PyMol Molecular Graphics System, Version 2 edu, Schrodinger, LLC). 

### 2.8. Statistical Analysis

GraphPad Prism 5 software (GraphPad, La Jolla, CA, USA) was used to compare the results of all tests. Statistically significant differences between groups were determined by one-way ANOVA and Tukey’s *post hoc* multiple comparison tests. All data are presented as mean ± SD. *p*-value < 0.05 was considered statistically significant.

## 3. Results

### 3.1. Purification and Characterization of the nLasB

**Figure 1:**
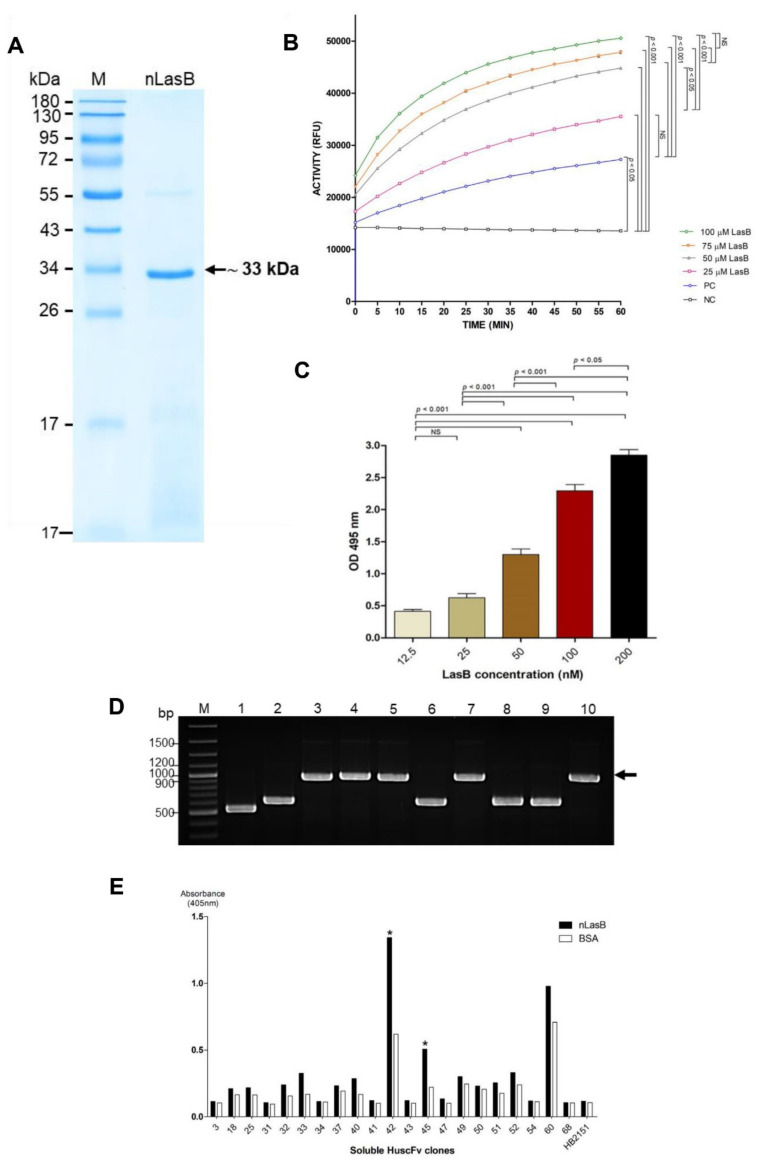
Preparation of native LasB (nLasB) and selection of the LasB-bound HuscFv-displaying phage clones. (**A**) Purified nLasB revealed by SDS-PAGE and CBB staining. M, protein molecular mass marker (ThermoFisher Scientific, Rockford, lL, USA); nLasB, purified native LasB (~33 kDa, arrow). Numbers at the left are protein masses in kDa. (**B**) Determination of enzymatic activities of the purified nLasB using fluorogenic substrate assay. PC, positive control which was porcine pancreatic elastase (0.025 U/mL) mixed with substrate; NC, negative control which was reaction buffer mixed with substrate; ACTIVITY (RFU), relative fluorescence units of the enzymatic activity per minute. (**C**) Results of elastin-Congo Red assay for determining elastolytic activity of the nLasB. NS, not significantly different. (**D**) Colony-PCR analysis of representative phage-transformed HB2151 *E. coli* clones; black arrow indicates amplicon of complete *huscfvs* (1000 bp) in the phage-transformed-*E. coli*. Smaller DNA bands are truncated *huscfvs*. M, DNA marker (Thermo Fisher Scientific); 1-10, the phage-transformed *E. coli* clones nos. 1-10, respectively. Numbers at the left are DNA masses in base pairs (bp). (**E**) Results of indirect ELISA for determining the binding of HuscFvs in lysates of phage-transformed HB2151 *E. coli* clones to nLasB, control BSA (antigen control), and HB2151 (lysate of original HB2151 *E. coli* without *huscfv*-phagemid) as background binding control; asterisks indicate the clones that their HuscFvs gave the high binding activity to nLasB (an ELISA signal at OD 405 nm to the nLasB: OD 405 nm to the control BSA was more than 2). These clones were selected for further experiments.

The nLasB was eluted from the DEAE-Sepharose Fast Flow column at 300 mM NaCl. The purified nLasB protein after SDS-PAGE and CBB staining appear as predominant band at apparent molecular weight 33 kDa (Figure 1A). The 33-kDa band and the faint 55-kDa band in the SDS-PAGE gel were verified by LC-MS/MS as the *P. aeruginosa* elastase and immunomodulatory metalloprotease, respectively (Supplementary Table S1).

### 3.2. Enzymatic Activities of the Native LasB

The enzyme activity of the purified nLasB was determined using EnzChek^TM^ elastase assay. The fluorescence intensity increased with the increasing amounts of the nLasB (Figure 1B). 

Figure 1C shows results of the elastin Congo Red assay for testing elastolytic activity of the nLasB. The results are conformed to those of the fluorogenic substrate assay. The absorbance at 495 nm (Congo Red released from digested substrate) increased in nLasB dose-dependent manner. The results of both assays indicated that the so-prepared purified nLasB retained the inherent elastolytic activities. Therefore, the enzymatically active nLasB was used as antigen for phage bio-panning for selecting HuscFv-displaying phage clones that bound to the nLasB and for studying the elastase neutralizing activity of the nLasB-bound HuscFvs. 

### 3.3. Selection and Characterization of LasB-Bound HuscFv-Displaying Phages

After single-round bio-panning with the active nLasB protein, a total of 118 phage-transformed *E. coli* clones that grew on the selective agar plates were subjected to colony PCR analysis for determining the HuscFv-coding genes (*huscfvs*). Among these clones, 45 clones (38.13%) were *huscfv*-positive as they yielded 1000-bp amplicons, indicating that they carried recombinant *huscfv*-phagemids. Representatives of the *huscfv*-positive clones (clones 3–5, 7, and 10) are shown in Figure 1D. The other clones (1, 2, 6, 8, and 9) in the same Figure carried truncated *huscfv* sequences. 

After the *huscfv*-positive HB2151, *E. coli* were grown under IPTG induction, lysates of 21 *huscfv*-positive clones could express soluble HuscFv proteins. By indirect ELISA, the HuscFvs of two phage-transformed *E*. *coli* clones (nos. N42 and N45) gave significant ELISA signals to nLasB (more than two times to the antigen control or BSA) and above the background control (HB2151, lysate of original HB2151 *E. coli* without *huscfv-*phagemid) (Figure 1E). The *huscfvs* of these two clones showed complete DNA sequences coding for VH-linker-VL (HuscFvs). Therefore, the *huscfvs* of the N42 and N45 *E. coli* clones were sub-cloned into plasmid vector for large-scale production of the HuscFvs.

### 3.4. Recombinant HuscFvs to LasB

**Figure 2:**
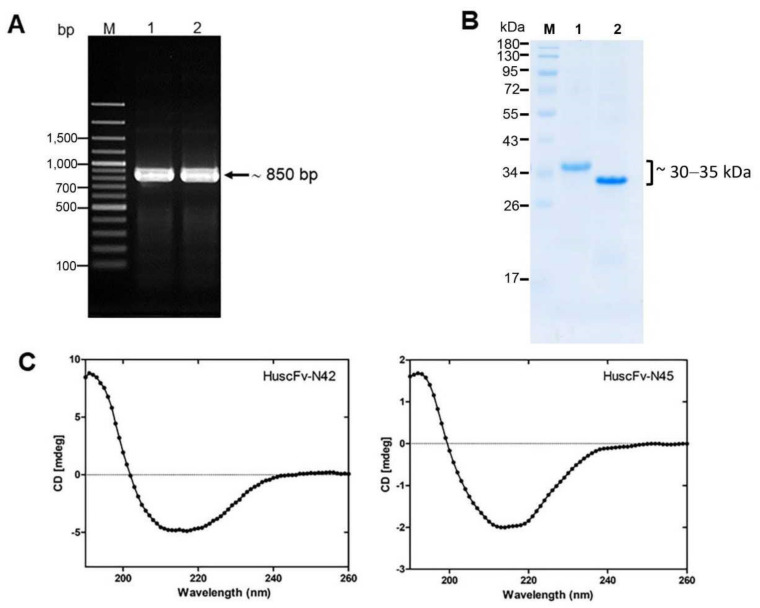
Production of recombinant LasB-bound HuscFvs. (**A**) Amplicons of *huscfv*-LIC fragments for sub-cloning into pLATE52 vector. M, 100-bp plus DNA marker; 1 and 2, *huscfv*-LIC amplicons of the transformed NiCo21(DE3) *E. coli* clones N42 and N45, respectively. Numbers at the left are DNA sizes in bp. (**B**) SDS-PAGE analysis of LasB-bound HuscFvs. M, protein marker; 1 and 2, purified HuscFv-N42 and HuscFv-N45, respectively (~30, 35 kDa). Numbers at the left are protein molecular masses in kDa. (**C**) CD spectra of the refolded HuscFv-N42 (left) and HuscFv-N45 (right).

After subcloning of the *huscfvs* of the *E. coli* clones N42 and N45 from recombinant pCANTAB-5E phagemids into the pLATE52 plasmids, the PCR products showed the bands at the expected size of around 850 bp (Figure 2A). The LasB-bound HuscFvs with the 6× His tag at the N-terminal and E-tag at the C-terminal were expressed from the *huscfv*-plasmid transformed NiCo21(DE3) *E. coli* under IPTG induction. Figure 2B shows SDS-PAGE-separated- and CBB-stained LasB-bound HuscFvs (~30, 35 kDa) after purification and refolding from the inclusion bodies of the N42 and N45 *E. coli* clones. From the program analysis of the CD spectra (Figure 2C), the refolded HuscFv-N42 and HuscFv-N45 were found to acquire mainly β-sheet structure, which is the characteristic of immunoglobulin molecule. Moreover, the refolded HuscFvs retained the nLasB binding ability, as demonstrated by indirect ELISA (Supplementary Figure S1), indicating correct folding of the antibody molecules. 

### 3.5. HuscFvs-Mediated Neutralization of the LasB Elastase Activity 

**Figure 3:**
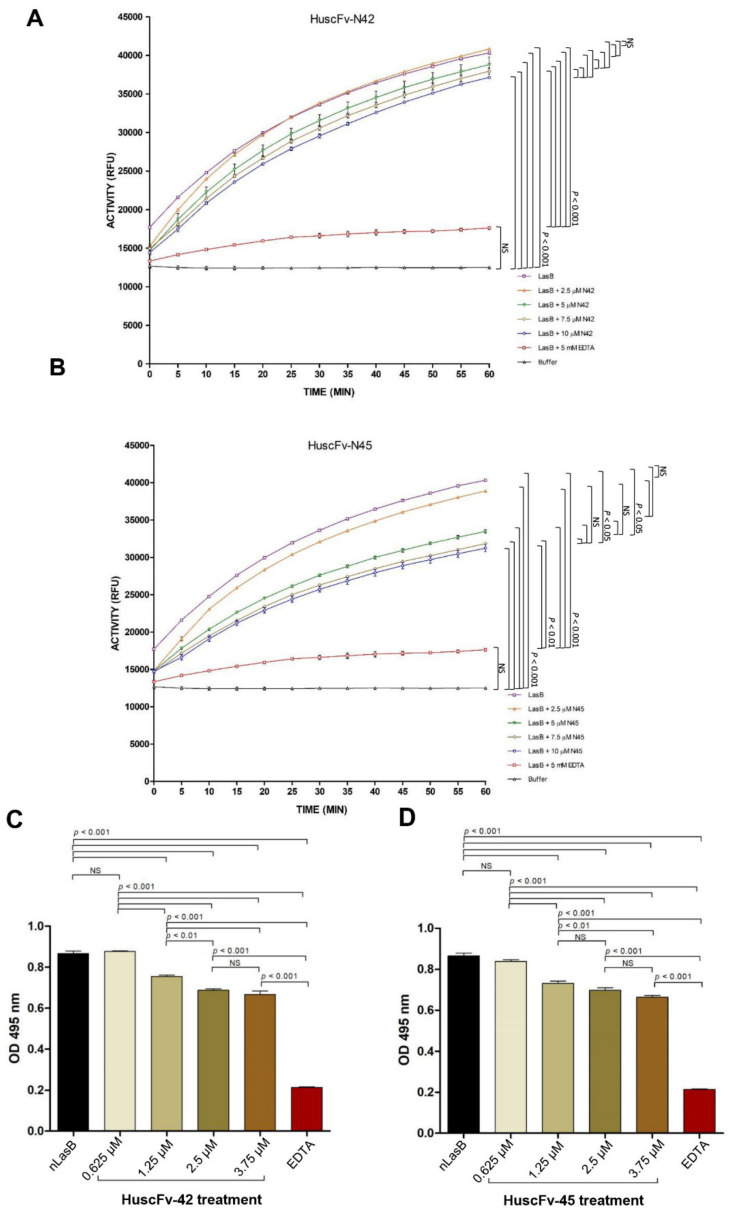
HuscFvs-mediated neutralization of LasB elastolytic activity. (**A**,**B**) The results of fluorogenic substrate assay shown as plots of elastase activity of LasB (negative neutralization control), nLasB after treatment with different concentrations of HuscFv-N42 (**A**) and HuscFv-N45 (**B**), compared to nLasB in Buffer (baseline) and nLasB treated with 5 mM EDTA (positive neutralization control). ACTIVITY (RFU), relative fluorescence units of the enzymatic activity per minute. NS, not significantly different. (**C**,**D**) are the results of elastin-Congo Red assay for determining HuscFvs-mediated neutralization of the nLasB-elastolyticity. The bar graphs of average (mean ± SD) of OD 495 nm of three independent experiments are shown. Elastolytic activities of the nLasB after treatment with various concentrations of HuscFv-N42 (**C**) and HuscFv-N45 (**D**), compared with that of LasB alone (negative neutralization control) and nLasB treated with 5 mM EDTA (positive neutralization control). NS, not significantly different.

The fluorogenic substrate assay was used to determine the neutralizing activity of the HuscFvs on the LasB elastolytic activity. The percentages of relative fluorescence units emitted from the DO-elastin substrate after the enzyme was treated with different concentrations of HuscFvs and controls are shown in Figure 3A,B. The fluorescence intensity of nLasB treated with HuscFv-N42 showed decreasing trend as the antibody concentrations increased, albeit not significantly different from the nLasB alone (without HuscFvs). The nLasB treated with the HuscFv-N45 had significantly decreased enzymatic activity as the HuscFv concentrations increased. The results of this assay indicate that the HuscFvs neutralized the nLasB enzymatic activity in a dose-dependent manner. In this experiment, the 5 mM EDTA which was used as positive LasB elastase neutralization control, effectively neutralized (abolished) the enzymatic activity.

By using elastin Congo Red assay, there was a significant suppression of LasB elastase activity after HuscFvs-treatment (decreased OD 495 nm of the released Congo Red from the Congo Red-elastin substrate), compared the negative neutralization control (native LasB alone) (Figure 3C,D). The suppression also showed the HuscFv concentration-dependent trend similar to the fluorogenic substrate assay. These results indicate that the HuscFv-N42 and HuscFv-N45 have inhibitory ability on the nLasB elastolytic activity. 

### 3.6. Predicted LasB Residues Bound by HuscFvs 

The 3D models of the HuscFv-N42 and HuscFv-N45 showed reliable Ramachandran plots (Supplementary Figure S2). The proportions of residues in the most favored regions, the additional allowed regions, the generously allowed regions, and the disallowed regions of the Ramachandran diagrams of HuscFv-N42 were 87.3, 10.8, 0.0, and 2.0% and those of the HuscFv-N45 were 90.2, 6.7, 1.5, and 1.5%, respectively. 

**Figure 4:**
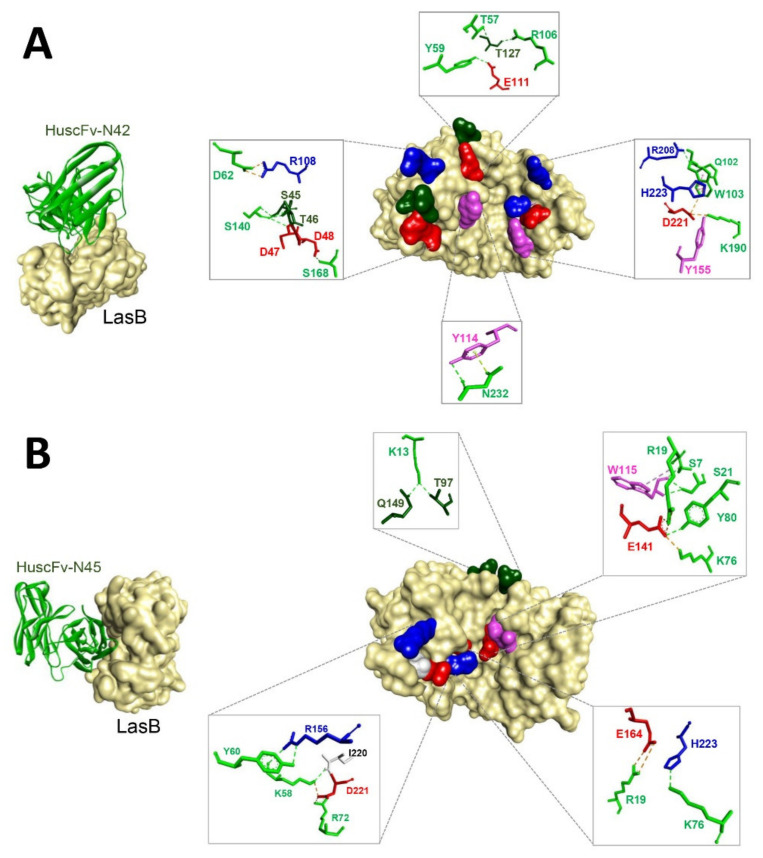
Presumptive binding of HuscFvs to LasB as predicted by computerized homology modeling and intermolecular docking. (**A**) LasB-HuscFv-N42 and (**B**) LasB-HuscFv-N45. Left panels of (**A**,**B**) show the presumptive interface contacts between the LasB (light brown) and the HuscFvs (green). Both antibodies interact with the LasB near or in the active/catalytic site. Right side of (**A**,**B**) shows contact residues of LasB with HuscFvs-N42 and HuscFv-N45, respectively. The LasB amino acids are colored according to the CINEMA color scheme: polar negative D and E, red; polar positive H and R, blue; polar neutral S, T, and Q, dark green; non-polar aromatic W and Y, magenta; and non-polar aliphatic I, white (grey in **B**, right panel).

The lowest local energies of the interactions between *Pseudomonas aeruginosa* LasB (elastase) and HuscFv-N42 and HuscFv-N45, based on the ClusPro docking server were −17.4 and −17.0 kcal/mol, respectively. Details of the LasB-HuscFv intermolecular docking including the interactive residues of the LasB and the HuscFv amino acids and domains as well as the intermolecular bonds between the two parties are shown in Table 1 and Figure 4. 

By the in silico docking, HuscFv-N42 was predicted to use VH-CDRs 2 and 3 and VL-CDRs 1–3, as well as the help from the peptide linker between the VH and VL domains, to form contact interfaces with the residues on the LasB target (right panel of Figure 4A and Table 1). There were 10 hydrogen bonds formed between the HuscFv-N42 and the LasB including S168 of VL-CDR1 with LasB D48; Y59 of VH-CDR2 with LasB E111; N232 of VL-CDR3 with LasB Y114; T57 of VH-CDR2 and R106 of VH-CDR3 with LasB T127; K190 of VL-CDR2 with LasB Y155; Q102 of VH-CDR3 with LasB R208; and S140 of the peptide linker with LasB S45, T46, and D47. Two salt bridge interactions were also demonstrated between HuscFv-N42 and the LasB including D62 of VH-CDR2 with LasB R108; and K190 of VL-CDR2 with LasB D221. A hydrophobic interaction (π-π stacking) between W103 of VH-CDR3 and H223 of the bacterial enzyme are also shown. 

The HuscFv-N45 was predicted to form an ionic bond with D221 of the LasB protease through K58 of VH-CDR2 and R72 of VH-FR3. The antibody also used VH-CDR2, VH-FR1, and VH-FR3 to form hydrogen bonds with the target antigen, i.e., K13 of VH-FR1 with LasB T97 and Q149; S7, R19, and S21 of VH-FR1 with LasB W115; K58 of VH-CDR2 with LasB R156 and I220; Y60 of VH-CDR2 with LasB R156; and K76 of VH-FR1 with LasB H223. Lysine 76 of VH-FR1 and Y80 of VH-FR3 formed a contact with LasB E141 via salt bridge interaction. The salt bridge bonding also occurred between R19 of VH-FR1 and E164 of the LasB (right panel of Figure 4B).

## 4. Discussion

LasB (elastase/pseudolysin) is one of the extracellular proteases secreted by *P. aeruginosa* in the infected host and in *in vitro* culture. This detrimental enzyme has multifunctional activities; it degrades a variety of the host proteins in tissues, extracellular matrices, as well as the host immune components for bacterial thriving, successful establishment of the infection, dissemination, and invasion [2]. Thus, it is an attractive target of innovative therapeutic agents for disarming the infecting *P. aeruginosa*. The lasB is encoded by *las*B gene and is produced by the bacteria (both planktonic and biofilm forming cells) as an enzymatically inactive pre-proenzyme (~55 kDa) with a classical signal peptide (2.4-kDa) and a covalently linked amino-terminal propeptide (18-kDa) [2,49,50]. The signal sequence is removed upon passage through the inner membrane into the periplasm, where the propeptide is rapidly cleaved off by autoproteolysis [51]. The propeptide also functions as an intramolecular chaperone required for correct folding and the LasB secretion competence [52,53]. The LasB translocates through the outer membrane by general secretory pathway (type II secretion system) into the bacterial milieu/culture medium as a 33-kDa mature and active protease [4]. In this study, the mature LasB was isolated from the culture supernatant of *P. aeruginosa*. The purified LasB preparation contained predominantly the 33-kDa mature protein, with a trace of the pre-proenzyme (55-kDa) and the 18-kDa amino-terminal propeptide (Figure 1A); the two latter should be from the lytic bacterial cells in the culture. By using fluorescent substrate and Congo Red assays, the purified nLasB showed enzymatic activity of which the kinetics of substrate cleavage determined by the former assay are, more or less, similar to those reported previously [54]. 

The active LasB/elastin was used as a bait (antigen) to fish out the HuscFv-dispalying phage clones from the previously constructed HuscFv phage display library by means of the conventional phage bio-panning process. Soluble HuscFvs recovered from the lysates of two *huscfv*-phagemid-infected HB2151 *E. coli* clones bound to the purified nLasB. Since the expression of the soluble HuscFvs from the *huscfv*-phagemids by the HB2151 *E. coli* is under a weak promotor (pLacZ), the amounts of the required HuscFvs were inadequate for further experiments. We, therefore, subcloned the *huscfvs* into pLATE52 expression plasmid and used the NiCo21(DE3) *E. coli* as the factory for large scale production of the HuscFvs. The *huscfv*- pLATE52 transformed *E. coli* produced high amount of the HuscFvs in the bacterial inclusion bodies; thus, purification and refolding of the antibodies had to be performed. After refolding of the recombinant antibodies, we checked them by circular dichroism and found that the proteins had high percentages of β-sheet structure, indicating that they were likely immunoglobulins. The purified refolded proteins showed molecular masses at ~30 and ~35 kDa (Figure 2B), which are the correct sizes of the single-chain antibodies that consists of VH-(Gly_4_Ser)_3_-VL. Besides, the refolded proteins bound to the nLasB as did the soluble HuscFvs derived from the *huscfv*-phagemid-transformed HB2151 *E. coli* clones N42 and N45, verifying that they are nLasB-bound HuscFvs. We, therefore, tested the HuscFv-N42 and HuscFv-N45 further for their ability to neutralize the elastrolytic activity of the nLasB using the EDTA as the positive inhibitor control. 

The HuscFvs from both *E. coli* clones were found to neutralize the nLAsB enzymatic activity to significant degrees in both *in vitro* assays (fluorescence substrate and Congo Red assays) in a dose-dependent manner. The HuscFv-N45 performed better than the HuscFv-N42 on the same concentration basis. The EDTA at 5 mM, which was used as positive nLasB neutralization control, effectively neutralized (abolished) the nLasB activity. Less amounts of the EDTA (in μM range) were not tested. There are possibilities to enhance the LasB neutralizing activity of the HuscFvs by either adjusting the antibody to target ratio in the *in vitro* assay such that the two reactants reach their equivalence zone. Moreover, target binding affinity of the antibodies may be increased by CDR resurfacing [55], *i.e.*, point mutation of some residues exposed on the CDR surface without changing the ligand binding specificities and the CDR conformations. It should be noted that the HuscFvs from the N42 and N45 *E. coli* clones had different molecular masses although the lowest energy that they used to form contact interfaces with the LasB target were relatively similar (−17.4 and −17.0 kcal/mol, respectively) as predicted by the computerized molecular docking. The molecular mass difference should be from the different numbers and sizes of amino acid residues that the two HuscFvs contained especially at the VH CDR3 which is the main target binding domain of antibody. VH CDR3 lengths in human ranged from 4 to 36 residues according to the IMGT numbering; the average length of VH CDR3 in human is 15.5 ± 3.2 amino acid residues and the most frequently occurring length in human VH CDR3 was 14 residues [56]. The difference in the molecular contents which leads to different sizes and conformations of the HuscFvs-tertiary structures should explain the difference in target binding specificity, affinity, and/or functional efficacy of the different HuscFvs.

Although the detailed mechanisms of the HuscFvs in mediating the protease neutralization requires elucidation by laboratory experiments, the computerized simulation indicated that the HuscFv-N42 and HuscFv-N45 formed contact interfaces with residues critical for LasB-catalytic activities. The LasB residues bound by the HuscFv-N42, *i.e.*, Y155, D221, and H223, are involved in substrate binding [41,57], while the HuscFv-N45 not only interacted with the substrate binding residues, D221 and H223, but also other amino acids, *i.e.*, E164, which is the important ligand of zinc co-factor of this metalloproteinase [41], and the E141 located in the center of the elastase catalytic site, which is pivotal for the LasB protease activity [41,58]. The binding to different residues of the target should explain the better enzyme neutralizing ability of the HuscFv-N45 than the HuscFv-N42. 

## 5. Conclusions

Engineered human monoclonal single-chain antibodies (HuscFvs) that neutralize the enzymatic activity of the *P. aeruginosa* LasB elastase were successfully generated using phage display technology. The HuscFvs should be tested further step-by-step towards the clinical use for mitigation of the disease severity caused by *P*. *aeruginosa* infection.

## Figures and Tables

**Table 1:** Presumptive residues of *P*. *aeruginosa* LasB (elastase) that were predicted by computer simulation to form the contact interface with residues and domains of the effective human single-chain antibodies, HuscFv-N42 and HuscFv-N45.

LasB Protein	HuscFv-N42		Interactive Bond (s)
Residue	Residue	Domain (s)	
S45	S140	Linker	Hydrogen
T46	S140	Linker	Hydrogen
D47	S140	Linker	Hydrogen
D48	S168	VL-CDR1	Hydrogen
R108	D62	VH-CDR2	Salt bridge
E111	Y59	VH-CDR2	Hydrogen
Y114	N232	VL-CDR3	Hydrogen
T127	T57/R106	VH-CDR2/CDR3	Hydrogen
Y155 (substrate binding)	K190	VL-CDR2	Hydrogen
R208	Q102	VH-CDR3	Hydrogen
D221 (substrate binding)	K190	VL-CDR2	Salt bridge
H223 (substrate binding)	W103	VH-CDR3	Hydrophobic (π-π stacking)
**LasB Protein**	**HuscFv-N45**		**Interactive Bond(s)**
**Residue**	**Residue**	**Domain(s)**	
T97	K13	VH-FR1	Hydrogen
W115	S7/R19/S21	VH-FR1	Hydrogen
E141 (located at the center of the catalytic site)	K76/Y80	VH-FR1/FR3	Salt bridge
Q149	K13	VH-FR1	Hydrogen
R156	K58/Y60	VH-CDR2	Hydrogen
E164 (ligand of zinc co-factor)	R19	VH-FR1	Salt Bridge
I220	K58	VH-CDR2	Hydrogen
D221 (substrate binding)	K58/R72	VH-CDR2/FR3	Ionic
H223 (substrate binding)	K76	VH-FR3	Hydrogen

## Data Availability

The data used to support the findings of this study are available from the corresponding author upon request.

## Supplementary Materials

The following data are available online https://www.mdpi.com/article/10.3390/pathogens10060765/s1, Figure S1: Ramachandran plots of the modeled HuscFv-N42 and HuscFv-N45; Table S1: LC-MS/MS of purified native LasB; Table S2: The predicted binding affinity (ΔG) and dissociation constant (Kd) of LasB bound by individual HuscFvs.
