# Supplementary material for: Human Single-Chain Antibodies That Neutralize Elastolytic Activity of Pseudomonas aeruginosa LasB
Source: Pathogens. 2021 Jun 17;10(6):765. doi: 10.3390/pathogens10060765 (PMC8234315; doi:10.3390/pathogens10060765)
Supplement: Supplementary file 1 [file pathogens-10-00765-s001.zip › pathogens-1252295-supplementary.pdf]

Supplementary Material

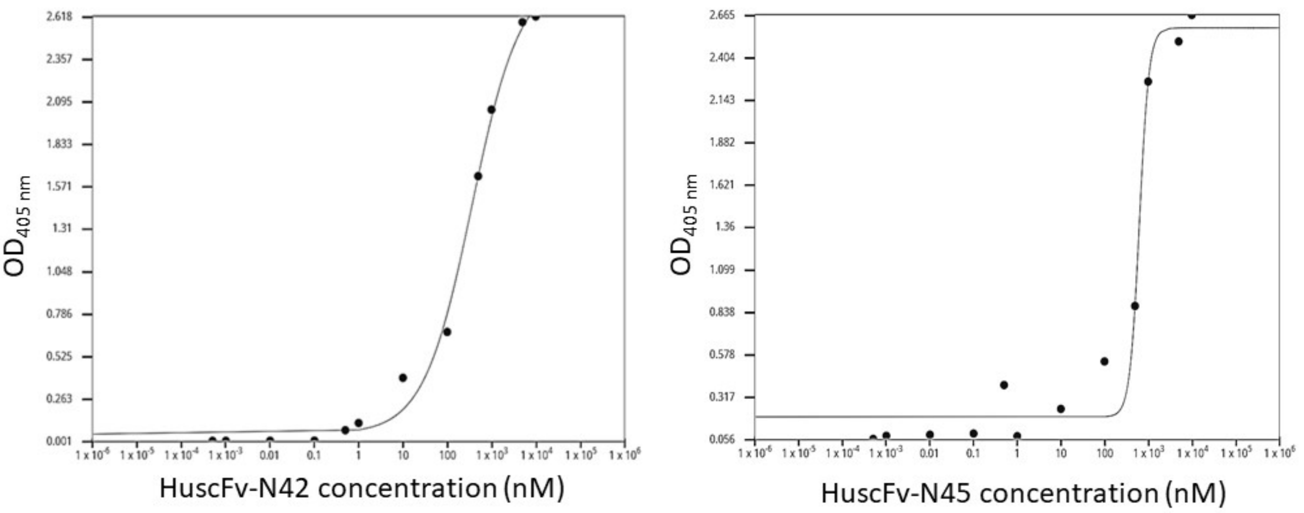

SUPPLEMENTARY FIGURE S1 | EC_{50} of refolded recombinant HuscFvs that bound to native LasB as determined by indirect ELISA. Serial concentrations of purified, refolded HuscFvs at 0.5-10 µM were incubated with immobilized nLasB (50 nM) in individual EIA/RIA wells. OD 405 nm signals were determined against blank (antigen-coated well incubated with diluent). The EC_{50} graphs of HuscFv-N42 and HuscFv-N45 were generated using EC50 Calculator (https://www.aatbio.com/tools/ec50-calculator).

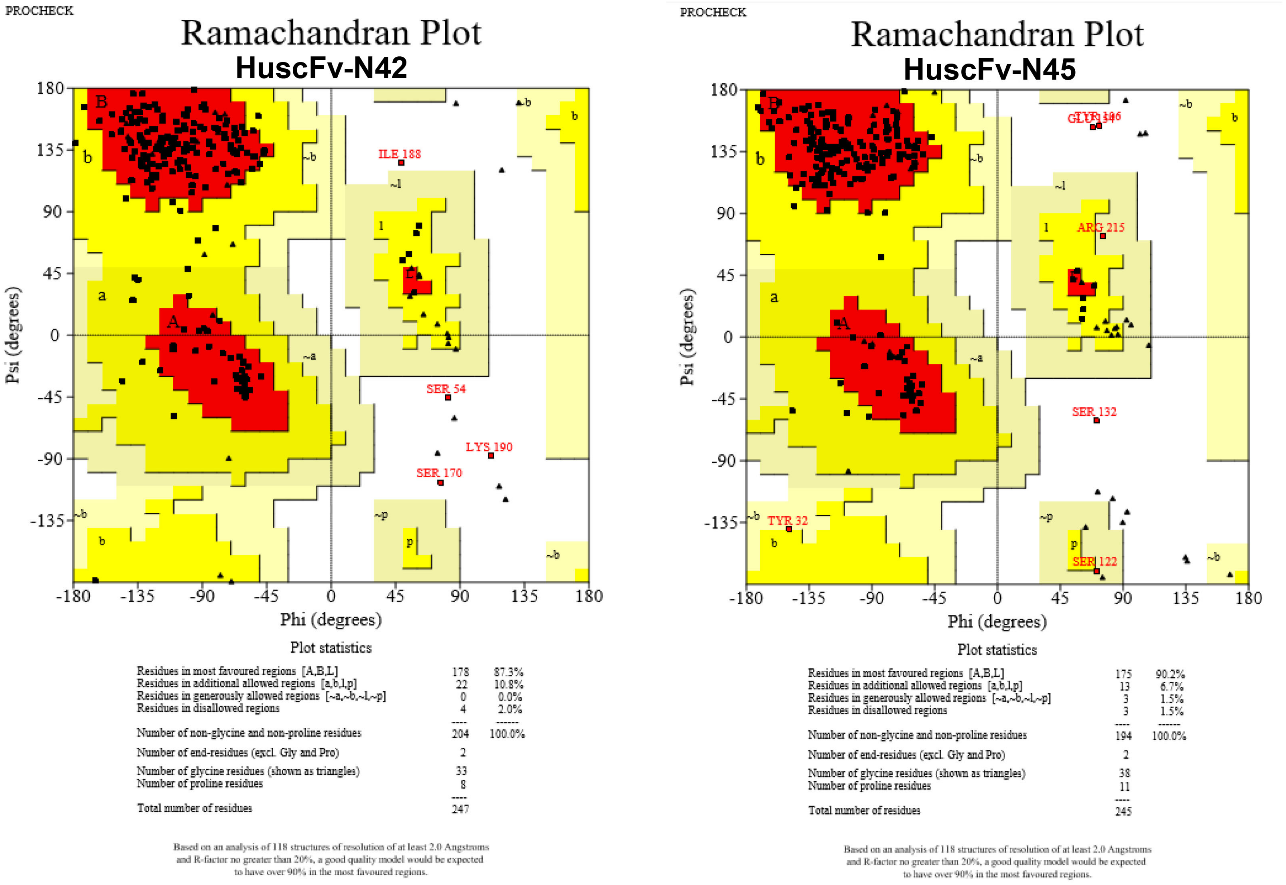

SUPPLEMENTARY FIGURE S2 | Ramachandran plots of the modeled HuscFv-N42 and HuscFv-N45. The most favored regions, additional allowed regions, generously allowed regions and disallowed regions are colored in red, yellow, light yellow, and white, respectively.

SUPPLEMENTARY TABLE S1 | LC-MS/MS Mascot results of peptides generated from in-gel tryptic digestion of the 33-kDa native LasB of *P. aeruginosa* searching against the NCBInr database.

Protein	Orthologous protein	Accession no.	Protein score	Peptide score	Matched peptide sequence
LasB (33 kDA)	Elastase OS=*Pseudomonas aeruginosa* (strain ATCC 15692 / DSM 22644 / CIP 104116 / JCM 14847 / LMG 12228 / 1C / PRS 101 / PAO1)	Gi 156255901	304	48.43	NYSAADVTR
				48.55	NYSAADVTR
				44.77	NYSAADVTR
				49.1	NYSAADVTR
				40.84	NYSAADVTR
				48.5	NYSAADVTR
				43.82	FACPTNTYK
				55.18	FACPTNTYK
				49.72	FACPTNTYK
				45.17	GKNDFLIGYDIK
				43.42	GKNDFLIGYDIK
				62.11	SAQNRNYSAADVTR
				55.43	SAQNRNYSAADVTR
				36.11	AFYLLANSPGWDTR
				51.79	AFYLLANSPGWDTR
				72.96	AFYLLANSPGWDTR
				64.87	AFYLLANSPGWDTR
				27.74	YTYGSDYGPLIVNDR
				55.55	AFYLLANSPGWDTRK
				41.06	AFYLLANSPGWDTRK
				58.23	AFYLLANSPGWDTRK
				53.97	IGKYTYGSDYGPLIVNDR
				41.58	SIDNASQYYNGIDVHHSSGVYNR
55 kDa protein	Immunomodulating metalloprotease OS=*Pseudomonas aeruginosa* (strain ATCC 15692 / DSM 22644 / CIP 104116 / JCM 14847 / LMG 12228 / 1C / PRS 101 / PAO1) OX=208964 GN=impA PE=1 SV=1	Gi Q9I5W4	73	50.86	TPAIQQECAAR
				53.21	EAGAAQKELGSYAGQR
				39.42	EAGAAQKELGSYAGQR

SUPPLEMENTARY TABLE S2 | The binding affinity (ΔG) and dissociation constant (Kd) predicted values of LasB bound to individual HuscFv.

LasB-HuscFv complex	Complex name	ΔG (kcal mol^{-1})	K_d (M) at 25.0 °C
LasB-N42	model.000.08	-15.7	3.10E-12
	model.000.09	-10.3	2.90E-08
	model.000.12	-13.3	1.70E-10
	model.000.15	-12.7	4.80E-10
	model.000.06	-12	1.60E-09
	model.000.07	-12.1	1.40E-09
	model.000.28	-12.5	6.80E-10
	model.000.11	-12	1.50E-09
	model.000.04	-14	5.60E-11
	model.000.22	-13	3.10E-10
	model.000.16	-9.1	2.20E-07
	model.000.23	-14.8	1.40E-11
	model.000.18	-14.1	4.80E-11
	model.000.05	-12.5	6.60E-10
	model.000.24	-13	2.80E-10
	model.000.10	-11.9	1.70E-09
	model.000.02	-17.4	1.70E-13
	model.000.19	-10.4	2.20E-08
	model.000.27	-11.5	3.80E-09
	model.000.01	-14.8	1.50E-11
	model.000.25	-13.7	8.30E-11
	model.000.13	-13.7	8.30E-11
	model.000.17	-11.6	2.90E-09
	model.000.00	-13.5	1.20E-10
	model.000.14	-15	9.50E-12
	model.000.21	-12.4	7.80E-10
	model.000.20	-12.2	1.10E-09
	model.000.26	-12.9	3.20E-10
	model.000.03	-12.9	3.40E-10

LasB-HuscFv complex	Complex name	ΔG (kcal mol^{-1})	K_d (M) at 25.0 °C
LasB-N45	model.000.08	-14.1	4.30E-11
	model.000.09	-12.3	9.40E-10
	model.000.12	-11.7	2.50E-09
	model.000.15	-11.2	6.40E-09
	model.000.06	-14	5.20E-11
	model.000.07	-10.7	1.40E-08
	model.000.28	-11.9	1.70E-09
	model.000.11	-11.9	1.80E-09
	model.000.04	-13.5	1.20E-10
	model.000.22	-14.7	1.70E-11
	model.000.16	-13.2	2.10E-10
	model.000.23	-7.6	2.70E-06
	model.000.18	-13.9	6.20E-11
	model.000.05	-12.6	6.20E-10
	model.000.24	-10.5	2.00E-08
	model.000.10	-10.9	9.70E-09
	model.000.02	-12.3	9.90E-10
	model.000.19	-14.5	2.30E-11
	model.000.27	-10.6	1.80E-08
	model.000.01	-11.5	3.60E-09
	model.000.25	-13.2	2.20E-10
	model.000.13	-14.5	2.20E-11
	model.000.17	-12.8	4.10E-10
	model.000.00	-13.1	2.50E-10
	model.000.14	-11	9.30E-09
	model.000.21	-12.1	1.30E-09
	model.000.20	-9.7	8.00E-08
	model.000.26	-14.1	4.50E-11
	model.000.03	-17	3.50E-13
